# *Daphnia magna* diapause-interrupted embryogenesis has changes in histone modifications at H3K9

**DOI:** 10.1186/s13072-025-00658-7

**Published:** 2026-01-24

**Authors:** Luxi Chen, Rocío Gómez, Frauke Brenne, Linda C. Weiss

**Affiliations:** 1 Department of Animal Ecology, Evolution and Biodiversity, Ruhr-University Bochum, Universitaetsstr. 150, 44780 Bochum, Germany; 2 Departament of Biology, Faculty of Sciences, Universidad Autónoma de Madrid, Madrid, 28049 Spain

**Keywords:** H3K9ac, H3K9me3, *kat2a*, *suv39h1*, Crustacean, Epigenetic regulation

## Abstract

As a key component of freshwater ecosystem, *Daphnia* survives environmental challenges by entering a state of developmental arrest known as diapause. Diapause is not simply a sudden halt in growth and development, but a genetically regulated program involving a sequence of coordinated developmental processes. Histone modifications, as crucial epigenetic mechanisms that regulate gene expression by altering chromatin structure, are hypothesized to play a significant role in diapause regulation. This study focuses on the acetylation (H3K9ac) and tri-methylation (H3K9me3) at the H3K9 site. Given the established roles of H3K9ac in transcriptional activation and H3K9me3 in transcriptional silencing, we hypothesize that these antagonistic modifications play regulatory roles in the diapause developmental program of *Daphnia*. Via immunocytochemistry, we observed a significant overall decrease in H3K9ac and an increase in H3K9me3 levels in *Daphnia magna* diapausing cells, suggesting transcriptional silencing of H3K9-related genes. Notably, however, a subset of cells retained high H3K9ac and low H3K9me3 levels, indicating localized transcriptional activity of H3K9 related genes that may be essential for maintaining diapause and facilitating developmental resumption once conditions improve. We also examined the expression of the H3K9ac-related acetyltransferase gene *kat2a* and the H3K9me3-related methyltransferase gene *suv39h1* via quantitative PCR. *kat2a* was highly expressed during active embryonic development (pre- and post-diapause) and maintained a basal level during diapause, potentially supporting the transcriptional activity of H3K9-related genes in selected cells. On the other hand, *suv39h1* transcript levels remained low from ovulation through the entire diapause period, making its contribution to the elevated H3K9me3 levels during diapause unclear. However, the increase in *suv39h1* expression toward the end of diapause suggests its potential role in diapause termination and subsequent post-diapause development. Our results illustrate dynamic changes in H3K9 modifications throughout the diapause developmental program in *Daphnia*, indicating their role in diapause regulation by modulating gene expression via alterations to the chromatin landscape.

**Supplementary Information:**

The online version contains supplementary material available at 10.1186/s13072-025-00658-7.

## Introduction

The genus *Daphnia*, widely distributed in freshwater systems worldwide, fulfills a critical ecological role as both a grazer regulating phytoplankton growth and a primary prey item for higher trophic levels. The life cycle of *Daphnia* is highly responsive to environmental fluctuations. Under favorable conditions, asexually produced clonal embryos, known as continuously developing embryos, develop directly within the mother’s brood pouch and are released as juveniles [13]. When environmental conditions worsen (e.g., reduced photoperiod, temperature variation), *Daphnia* produces embryos that enter a suspended developmental state, referred to as diapause. These are termed diapause-interrupted embryos. In most *Daphnia* species, diapause-interrupted embryos are generated through sexual reproduction. Females first produce genetically identical males via canonical parthenogenesis and then produce haploid eggs via meiosis. These eggs require fertilization by the haploid spermatozoa meiotically produced by the males. In some *Daphnia* species, diapause-interrupted embryos also arise through asexual reproduction, by passing the need for fertilization [20, 21, 28, 29, 34). Regardless of the reproductive method, these embryos are encased in a thickened structure called the ephippium within the female’s carapace [28]. In this state, the embryos can suspend development for years or even decades, resuming embryonic growth when conditions again become favorable [9, 23]. Diapause, defined by a suspension of growth and development, entails a complex suite of biological and physiological adaptations governed by precise gene expression regulation [11, 16, 36, 54, 66]. Recent research increasingly highlights that shifts in gene expression are central to the initiation, maintenance, and termination of diapause, with an expanding focus on epigenetic mechanisms that orchestrate these processes throughout the diapause program. Epigenetic mechanisms, (e.g. DNA methylation, histone post-translational modifications, and non-coding RNA) modulate gene expression without altering the underlying DNA sequence, providing organisms with adaptive responses to environmental stresses. Among these, histone post-translational modifications are of particular interest in diapause research due to the high conservation of histone sequences, as well as histone gene expression and their modification systems across higher eukaryotes [1, 45, 50, 61, 89]. The reversible nature of histone post-translational modifications makes them particularly suited to orchestrating global shifts in gene expression, which is an essential requirement for organisms cycling between active development and suspended animation. By altering histone acetylation, and methylation states, cells can rapidly repress or activate large suites of genes in response to environmental cues associated with the onset, maintenance, or termination of diapause. Therefore, in this study, we focus on histone modifications to unravel an epigenetic logic underlying diapause transitions, aiming to clarify how these marks may integrate developmental and environmental signals to enable survival and adaptability during periods of environmental stress [5, 66].

Numerous studies have documented alterations in histone modifications across various sites and species (Table 1). Notably, research in *Artemia* demonstrates that aberrant histone modifications, such as H4K20me3, can obstruct the formation of diapause embryos, underscoring the importance of histone modifications in regulating the diapause developmental program [10]. Based on these findings, we propose that histone modifications play a critical role in supporting the diapause process in *Daphnia* diapause-interrupted embryos, encompassing preparation for developmental arrest, maintenance of the arrested state, and subsequent reactivation.

In this study, we focused on histone H3 and its specific lysine residues. Lysine, a positively charged amino acid, interacts strongly with the negatively charged DNA backbone. Acetylation neutralizes this charge, reducing histone-DNA affinity and loosening chromatin for better access by transcription factors and RNA polymerase, and other components of the transcriptional machinery. Conversely, lysine methylation is recognized by heterochromatin Protein 1 (HP1), promoting tighter nucleosome packing and increasing chromatin inaccessibility. These distinct chemical characteristics of lysine make it a critical site for modifications that regulate chromatin configuration and gene activity. Therefore, acetylation of H3 at lysine 9 (H3K9ac) marks active transcription, while trimethylation at the same site (H3K9me3) is linked to transcriptional repression [3, 26, 32].

Additionally, the enzymes modulating H3 lysine residues are well characterized. KAT2A (Lysine acetyltransferase 2 A) acetylates multiple positions on histone H3, including H3K9. KAT2A can also acetylate histones at enhancer regions, facilitating the recruitment of mediator complexes and other factors involved in enhancer-promoter communication [19, 25, 49, 77]. Conversely, once bound to H3K9 Lysine residues, HP1 can recruit histone methyltransferases like Suv39h1 (Suppressor of variegation 3–9 homolog 1) which further methylates adjacent histones, spreading the repressive mark and reinforcing the heterochromatin state. The genes *kat2a* and *suv39h1* were selected for this study due to their pivotal roles in H3K9 modification and high evolutionary conservation. Protein BLAST analysis confirmed strong conservation of their encoded protein sequences across diverse species, including *Homo sapiens*, *Mus musculus*, *Drosophila*, and *Daphnia magna* (Supplementary Material), supporting their investigation in *Daphnia* diapause as a conserved regulatory mechanism.

In this study, we assessed the levels of H3K9ac and H3K9me3 via immunocytochemistry (ICC) and the expression of kat2a and suv39h1 using reverse transcription quantitative polymerase chain reaction (RT-qPCR) in diapause-interrupted and continuously developing embryos. We hypothesized a reversible transition between a permissive (high kat2a/H3K9ac) and a repressive (high suv39h1/H3K9me3) epigenetic state, thereby correlating with developmental arrest and reactivation in *Daphnia*.

**Table 1:** An overview of study regarding histone modification during diapause across species

Species	Stage of diapause	Histone modification	References
*Austrofundulus limnaeus*	Embryonic diapause	H3K4 mono-/di-/tri-methylation	[81]
		H3S10 phosphate	
		H3K27 mono-/di-/tri-methylation	
*Artemia spec*	Embryonic diapause	H3K4 mono-/di-/tri-methylation	[10]
		H3K9 mono-/di-/tri-methylation	
		H3S10 phosphate	
		H3K27 mono-/di-/tri-methylation	
		H3K36 mono-/di-/tri-methylation	
		H3K79 mono-/di-/tri-methylation	
		H4K20 mono-/di-/tri-methylation	
*Artemia spec*	Embryonic diapause	H3S10 phosphate	[93]
		H3K56 acetylation	
*Dianemobius nigrofasciatus*	Embryonic diapause	H3S10 phosphate	[80]
		H3K14 acetylation	
*Ostrinia furnacalis*	larval diapause	H3K9me3	[43]
		H3K27me3	
*Caenorhabditis elegans*	larval diapause (dauer)	H4K20 di-/tri-methylation	[14]
*Helicoverpa armigera*	Pupal diapause	H3K4ac	[42]
		H3K9ac	
		H3K14ac	
		H3K18ac	
		H3K23ac	
		H3K36ac	
		H3K122ac	
*Drosophila melanogaster*	reproductive diapause	H3K4 trimethylation	[18]
		H3K9me3	
		H3k9ac	
		H3K27ac	
		H3K27 trimethylation	
		H3K36 monomethylation	

## Materials and methods

### Animal culture

*D. magna* clone Elias (collected by L. Weiss and R. Tollrian) and *D. magna* clone FT442 (kindly provided by Dieter Ebert) were cultured in a clone-specific manner at a constant temperature of 20 °C ± 0.1 °C in temperature- and photoperiod-controlled incubators (KB 750, Binder, Germany) in 1 L glass jars (WECK^®^, Germany).

Based on our previous work [7], we collected continuously developing embryos from *D. magna* clone FT442, where *Daphnia* females were kept under long-day photoperiod (16:8 h light: dark cycle) at a low density of 6 individuals/L at 20 °C ± 0.1 °C to prevent the produce of male offspring.

Since *D. magna* clone FT442 does not produce diapause-interrupted embryos via parthenogenesis, we collected these embryos by co-culturing clone FT442 with clone Elias to prevent inbreeding effects, inducing their formation through crowding and a shortened photoperiod. Specifically, thirty females of clone FT442 together with twenty males of clone Elias were co-cultured in one 1 L glass jar under short-day photoperiod (8:16 h light: dark cycle) at 20 °C ± 0.1 °C. To collect male offspring, *D. magna* clone Elias females were kept under short-day photoperiod at a higher density (20 individuals/L) at 20 °C ± 0.1 °C. Males were identified by their large first antennae under stereomicroscope (SZX12, Olympus).

All animals were raised in an artificial *Daphnia* medium (ADaM, [35] and fed *ad libitum* daily with the green algae *Acutodesmus obliquus* suspension (> 1.5 mg C/L). The culture medium was refreshed weekly. Offspring, remnants, and exuvia were removed regularly using glass pipettes.

### Maintenance and resurrection of diapause-interrupted embryos

After the ephippia were cast off during the mothers‘ molt, they were collected within 24 h and stored at 4 °C in the absence of light to maintain diapause until further use. Since the presence or absence of ephippia does not influence the hatching of *Daphnia* diapausing embryos, the embryos were carefully dissected from the ephippia using fine forceps for clearer observation of hatching [51, 58]. The dissected embryos were then transferred to 24-well clear flat-bottom cell culture plates (VWR, Germany) filled with 2 mL sterile ADaM medium. The plates were placed under constant photoperiod (simulated by fluora and biolux lamps, Osram, Germany) at 23 °C ± 0.1 °C. Hatching was checked daily with a stereomicroscope. Sterile ADaM medium was replaced every other day.

### Staging of *D. magna* continuously developing and diapause-interrupted embryos

**Fig. 1:**
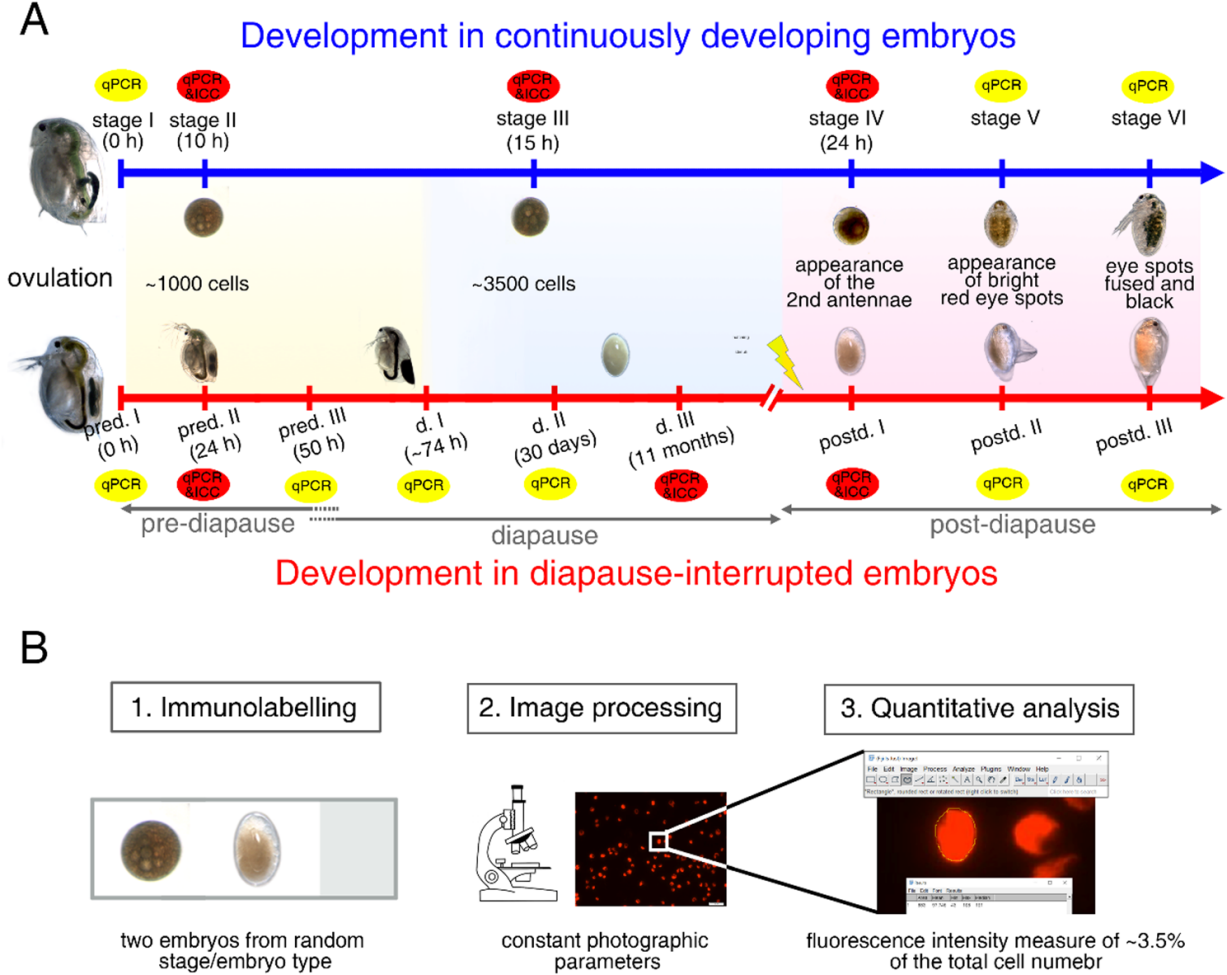
Experimental design and analytical workflow. Schematic timeline illustrating the experimental sampling stages for ICC and RT-qPCR analysis (**A**). Flowchart depicting the steps involved in the semi-quantitative measurement process (**B**)

*Daphnia* females capable of producing continuously developing and diapause-interrupted embryos can be distinguished by ovary morphology. Females capable of producing continuously developing embryos were cultured individually in 50 mL vials with 40 mL ADaM, while females capable of producing diapause-interrupted embryos were co-cultured with one male *D. magna* clone Elias in the same conditions. Ovulation was monitored at 15-minute intervals. All animals were maintained at 20 °C and under the original light conditions. Based on our previous studies, we established a comparative system to assess the differences between the two developmental pathways [7, 8]. Embryos were collected based on developmental time or embryo characteristics (Fig. 1).

We divided the embryogenesis of continuously developing embryos into six distinct stages. Stage I marked the onset of ovulation. Stage II was reached when the embryos had developed to 1000 cells, collected 10 h post-ovulation. Stage III was defined when embryos reached 3500 cells, collected 15 h post-ovulation. We here defined stage IV by the formation of antennal buds, collected around 24 h post-ovulation. Stage V was marked by the formation of two red eye spots, whereas stage VI was characterized by the fusion of these two eye spots into a single black eye spot (Fig. 1A).

For the embryogenesis of diapause-interrupted embryos, we identified nine distinct stages. The timepoint of ovulation was defined as pre-diapause (I) Pre-diapause II, occurring 24 h after ovulation, was characterized by high mitotic activity and approximately 1000 cells, corresponding to the cell number at stage (II) Pre-diapause III marked the end of pre-diapause phase, when mitotic activity halted at 50 h post ovulation, and the cell count stabilized at around 3500. After the pre-diapause III, embryos entered the diapause phase. The ephippium was naturally shed around 74 h post ovulation, embryos collected within 24 h after shedding were defined as being at diapause I. Following the shedding, embryos within the ephippia stored at 4 °C for 30 days were categorized as diapause II, while those kept for 11 months were classified as diapause (III) When exposed to suitable hatching stimuli, diapausing embryos exited diapause and transitioned into the post-diapause phase, though the exact transition timing is difficult to determine. We here defined post-diapause I by the formation of antennal buds, corresponding to the stage (IV) Post-diapause II was identified by the appearance of two red eye spots, while post-diapause III was characterized by the formation of a fused black eye spot, corresponding to stage V and stage IV respectively (Fig. 1A).

At the designated developmental stages, embryos were carefully dissected from the ephippium or *Daphnia’s* brood chamber using fine forceps. Specifically, diapause III embryos were removed from the ephippium under red light at 14 °C to prevent hatching [12]. Embryos were collected from separate *Daphnia* mothers or ephippia, three independent replications were performed. Three representative stages were chosen for ICC in both continuously developing and diapause-interrupted embryos to monitor the level of H3K9ac and H3K9me3. For a more detailed analysis of gene expression related to the enzymes responsible for H3K9ac and H3K9me3, RT-qPCR analysis was performed on six stages in continuously developing embryos and nine stages in diapause-interrupted embryos (Fig. 1A).

### Immunocytochemistry (ICC)

#### Immunolabelling

The collected embryos were fixed immediately in freshly prepared 4% PFA-TX (formaldehyde 37%; Merck, Germany; diluted in PBS: phosphate buffered saline 0.1 M, pH 7.4; with 0.05% Triton X; Serva, Germany) for an instant immunolabelling. We used polyclonal antibodies against H3K9ac (ab61231, Abcam, Germany) and H3K9me3 (ab8898, Abcam, Germany), both antibodies raised in rabbits. The specific sub-nuclear distribution of these two antibodies (same antibody product) was examined [24, 26]. To validate the specificity of our immunolabelling results, negative controls were performed using only the secondary antibody.

Immunolabelling was performed as previously described [7, 8], but with modifications to enable subsequent semi-quantitative analysis. The application of antibodies and all staining processes were kept consistent across all samples. For this purpose, after 15 min fixation in 4% PFA-TX, embryos from two different developmental stages or different embryo types were carefully squashed onto a single poly-lysine coated object slides (VWR, Germany) with coverslips. The positions of the embryos on the slide were documented, along with their corresponding stages and types (Fig. 1B).

The slides were then incubated with primary antibody (either H3K9ac or H3K9me3) diluted in PBS at an exact dilution ratio for exactly 2 h at room temperature (20 °C). For H3K9ac, the dilution rate was 1:30, while for H3K9me3, it was 1:120. Following three 5 min washes in PBS, the slides were incubated with goat anti-rabbit IgG (Alexa 594, Dianova Germany, from the same lot) at an exact dilution ratio of 1:150 in the dark for 1 h at room temperature. These dilution ratios were pre-tested to ensure uniform detection across all stages, especially those with low levels of H3K9ac and H3K9me3. Slides were then rinsed 3 times in PBS for 5 min each and subsequently mounted and cover-slipped in DAPI-Vectashield (H-120, Vecta Laboratories, Burlington USA) shielded from light. The coverslips were sealed with rapidly solidifying nail varnish. The Slides were kept at 4 °C in the dark.

#### Microscopy and image analysis

Each slide was photographed within 24 h after the staining process was completed. To minimize photobleaching effects, photography was done within 30 min, and the slides were not further used for semi-quantitative analysis. Immunofluorescence images were captured using a fluorescence microscope Axiophot, Zeiss (Germany) together with an XC10 monochrome digital camera and CellSense (Olympus, Germany). Since the levels of H3K9ac and H3K9me3 vary throughout the cell cycle [59]. We here focused solely on the analysis of interphase cells in the subsequent semi-quantitative analysis. Mitotic cells, particularly those from promtetaphse to telophase, were excluded based on DAPI staining and assessment of nuclear size and shape. All photographic parameters, including the exposure time (1.099s) and amplification factor (11.5 dB), were kept constant. All immunolabelling and documentation were performed within one month. In this way, differences resulting from the staining and photographic process were minimized. Given that the immunolabeling and photographic processes were well-controlled, the fluorescence intensity of a selected region should be relatively proportional to the amount of antigens, which further indicates the transcription level [33, 52, 56, 90, 92].

We aimed to compare the level of H3K9ac and H3K9me3 across different developmental stages and embryo types. Given the large variations in cell counts among these stages, for instance, there are over 7000 cells in post-diapause I and stage IV embryo, we randomly selected approximately 3.5% of the total cells count from each stage for semi-quantitative anlaysis. Specifically, we photographed 35 interphase cells in both pre-diapause II and stage II embryos, 120 interphase cells in diapause III and stage III embryos, and 245 interphase cells in post-diapause I and stage IV embryos for the semi-quantitative measurement [7]. The labeling intensity (mean gray values of the pixels in the measured area) of the entire nucleus was measured using the Measure-Function of the public domain software ImageJ (http://fiji.sc/Fiji) (Fig. 1B).

#### Statistical analysis for ICC

Results were analyzed with R 4.4.0 (R Core Team. [63] within RStudio [70]. Plots were generated with the package “ggplot2” [86]. Due to differences in sample size between stages and the lack of normal distribution, we used Kruskal-Wallis testing followed by Bonferroni’s post-hoc test to assess differences in intensity level between stages.

### Reverse transcription quantitative polymerase chain reaction (RT-qPCR)

The collected embryos were transferred to 1.5-ml Eppendorf tubes (Sarstedt, Germany), snap-frozen in liquid nitrogen, and stored at −80 °C ± 0.1 °C until RNA extraction. All samples were collected and processed within the same period. Three sets of biological replicates were collected for each developmental stages, with each containing 50 embryos.

#### RNA isolation

The embryos were manually homogenized in lysis buffer using a sterile pistil, and RNA extraction was performed using Quick-RNA MicroPrep-Kit (Zymo Research, USA) according to the manufacturer’s instructions. RNA Quantity was confirmed using an Eppendorf BioPhotometer (Eppendorf, Germany), while RNA integrity index was checked using an Agilent Tape Station (Agilent, Germany). Samples with an RNA integrity number > 8.0 were utilized. RNA concentration was determined using Qubit RNA broad range Assay Kit (Thermo Scientific, Germany).

#### Primers

*kat2a* and *suv39h1* primer pairs were designed based on their respective gene sequences (Dapma7bEVm004575t1 and Dapma7bEVm007233t1) retrieved from wfleaBase. Protein BLAST analysis confirmed that these sequences encode putative orthologs of the histone modifying enzymes KAT2A and SUV39H1, which are well-established writers for H3K9ac and H3K9me3, respectively. Primers were designed using Primer3 (http://frodo.wi.mit.edu/primer3/, Table 2). Primer pair for the gene *tbp* was obtained from [31].

**Table 2:** Primers pairs used in the qPCR analysis

Gene	Gene ID/wfleaBase	Primer sequences (5‘−3‘)	Annealing T (°C)	Products size (bp)	Amplification efficiency
*kat2a*	Dapma7bEVm004575t1	F: CGGCTACCACCAGAGCGACG R: TGGTGATTTGCGGGGCGATG	67 °C/ 63 °C	137	87%
*suv39h1*	Dapma7bEVm007233t1	F: GCATCGACGGTCGCAAGACA R: GGTCCACAACGGCACCGACT	58 °C/ 60 °C	130	85%
*tbp*	Dapma7bEV m004355t1	F: TGTGAGTCAGAACG AGACCT R: AAGAAGATTCAA GATTAGCAGCCC	46 °C/ 54 °C	120	86%

#### One step RT-qPCR

qPCR was performed with the Luna Universal One-Step RT-qPCR kit (New England Biolabs, Germany) following the manufacturer’s instructions and in a Light Cycler96 (Roche, Germany). PCR reaction was performed in a 10 µl volume consisting of 1 µl of 10 ng/µl RNA, 0.8 µl of 10 µM gene-specific primer pairs (Table 2), 5-µl reaction mix (2×), 0.5-µl RT enzyme mix, and 2.7-µl nuclease-free water. PCR reactions were carried out in 96-well plates (Sarstedt, Germany), which were incubated at 55◦C for 600 s and 95◦C for 60 s, followed by 45 cycles at 95◦C for 10 s and at 60◦C for 30 s. Melting curve analysis confirmed the specificity of the PCR products. Each plates included two technical duplicates, non-template controls (RNA replaced with water), and reverse transcription controls (reverse transcriptase was substituted with water). To ensure consistency across multiple plates, two standard RNA samples were amplified with the reference gene *tbp* on every plate. The variation in Cqs between these standards across all individual plates was less than 0.2%.

#### Statistical analyses for RT-qPCR

The stability of the reference gene between all stages and between the two embryo types was confirmed using “RefFinder” [87]. *tbp* was selected as the reference gene due to its high stability over all embryonic stages. The primer efficiency was calculated using the program “LinReg” [72]. The expression fold change of the target group was calculated based on [41]. For visualization, gene expression was log2 transformed and normalized to a standard stage (stage I and pre-diapause I). The statistical tests, including normality assessment using the Shapiro-Wilk test and one-sample t-tests, were performed in RStudio. Final results were visualized and generated using the “ggplot2” package [71].

## Results

### Subcellular distribution of H3K9ac and H3K9me3 in diapause-interrupted and continuously developing embryos

**Fig. 2:**
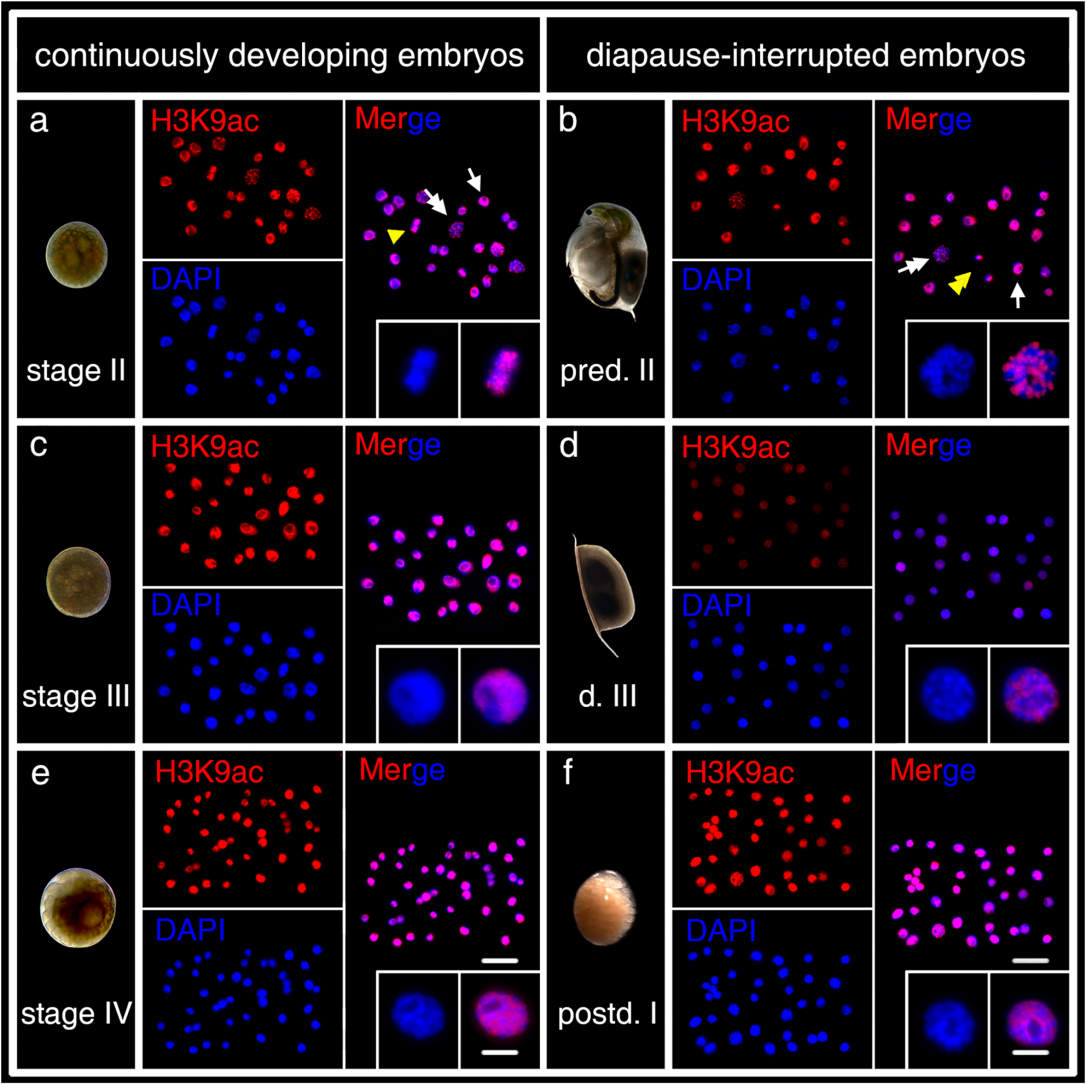
H3K9ac expression in continuously developing- (**a**, **c**, **e**) and diapause-interrupted embryos (**b**, **d**, **f**) of *D. magna*. H3K9ac is stained in red and chromatin with DAPI in blue. H3K9ac signals are predominantly localized to the DAPI-stained regions and are present throughout various stages of the cell cycle, like interphase (white arrow), prometaphase (white double arrow), and metaphase (yellow arrowhead), and anaphase/telophase (yellow double arrowhead). H3K9ac signals are observed in both active and diapausing cells. Magnified inset panels (DAPI and Merge) showed representative cells of each stage. Scale bars: zoom range = 5 μm; overview = 25 μm

**Fig. 3:**
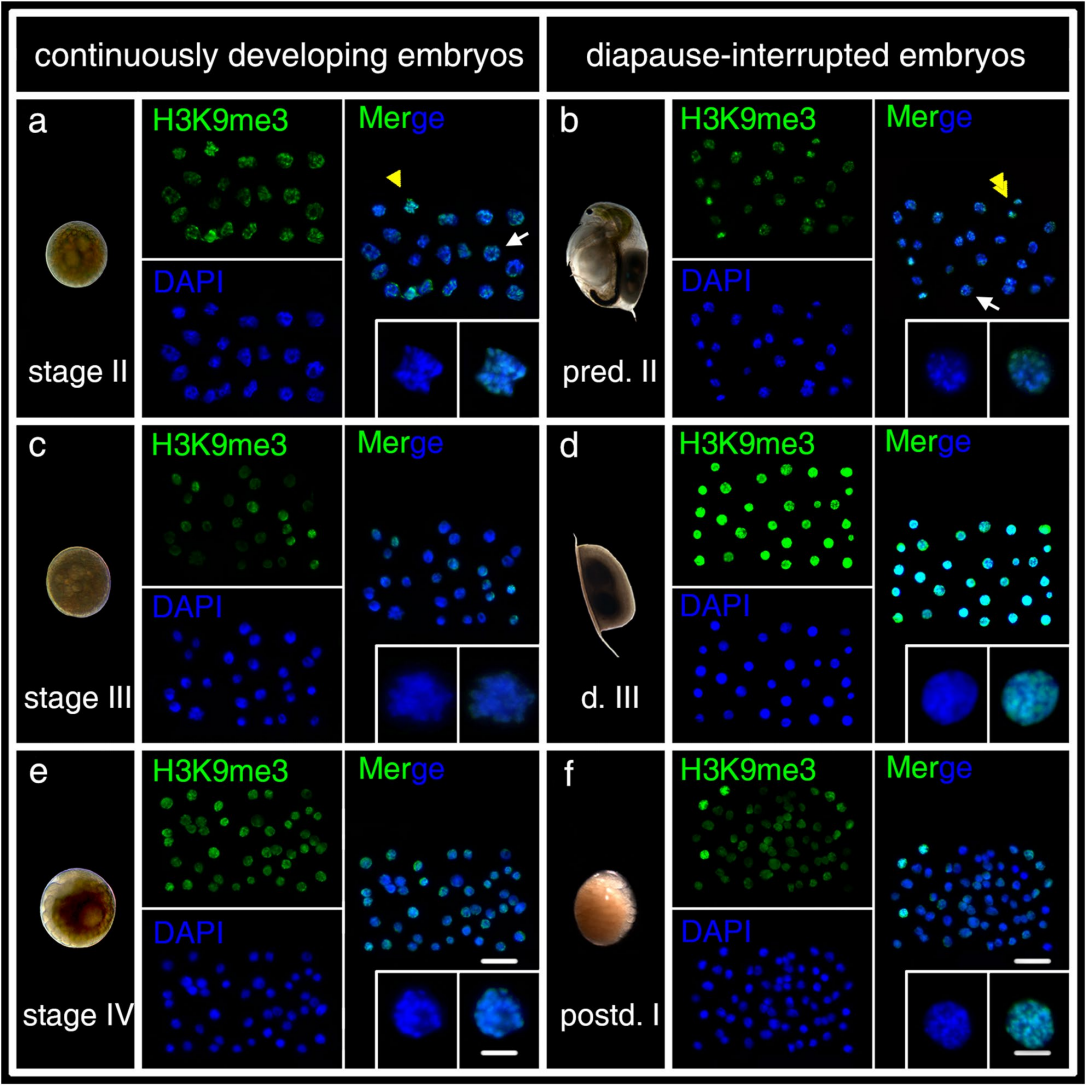
H3K9me3 expression in continuously developing- (**a**, **c**, **e**) and diapause-interrupted embryos (**b**, **d**, **f**) of *D. magna*. To clearly distinguish between the staining of H3K9me3 and H3K9ac in different figures, the H3K9me3 signals were displayed in green using Photoshop for enhanced visualization. Chromatin is stained with DAPI in blue. H3K9me3 signals are predominantly localized to the DAPI-stained regions and are present throughout various stages of the cell cycle, like interphase (white arrow), early anaphase (yellow arrowhead), and anaphase/telophase (yellow double arrowhead). H3K9me3 signals are observed in both active and diapausing cells. Magnified inset panels (DAPI and Merge) showed representative cells of each stage. Scale bars: zoom range = 5 μm; overview = 25 μm

H3K9ac and H3K9me3 signals are consistently located within the nucleus, independently of developmental stages or embryo types. The nucleolar regions, primarily composed of RNA and proteins, do not exhibit signals for H3K9ac or H3K9me3 (Figs. 2 and 3, white arrow). H3K9ac signals presumably localize to the less condense chromatin regions, as they overlap with areas of lower DAPI intensity labeling (Fig. 2d, e, f, magnifying inset panel), while H3K9me3 signals are found at condensed chromatin regions (Fig. 3d, e, and f, magnifying inset panel). Mitotic chromosomes also show clear signals of both H3K9ac and H3K9me3 (Fig. 2a, b, magnifying inset panel; Fig. 3a magnifying inset panel).

### Fluorescence intensity of H3K9ac during the embryonic development in continuously developing and diapause-interrupted embryos

**Fig. 4:**
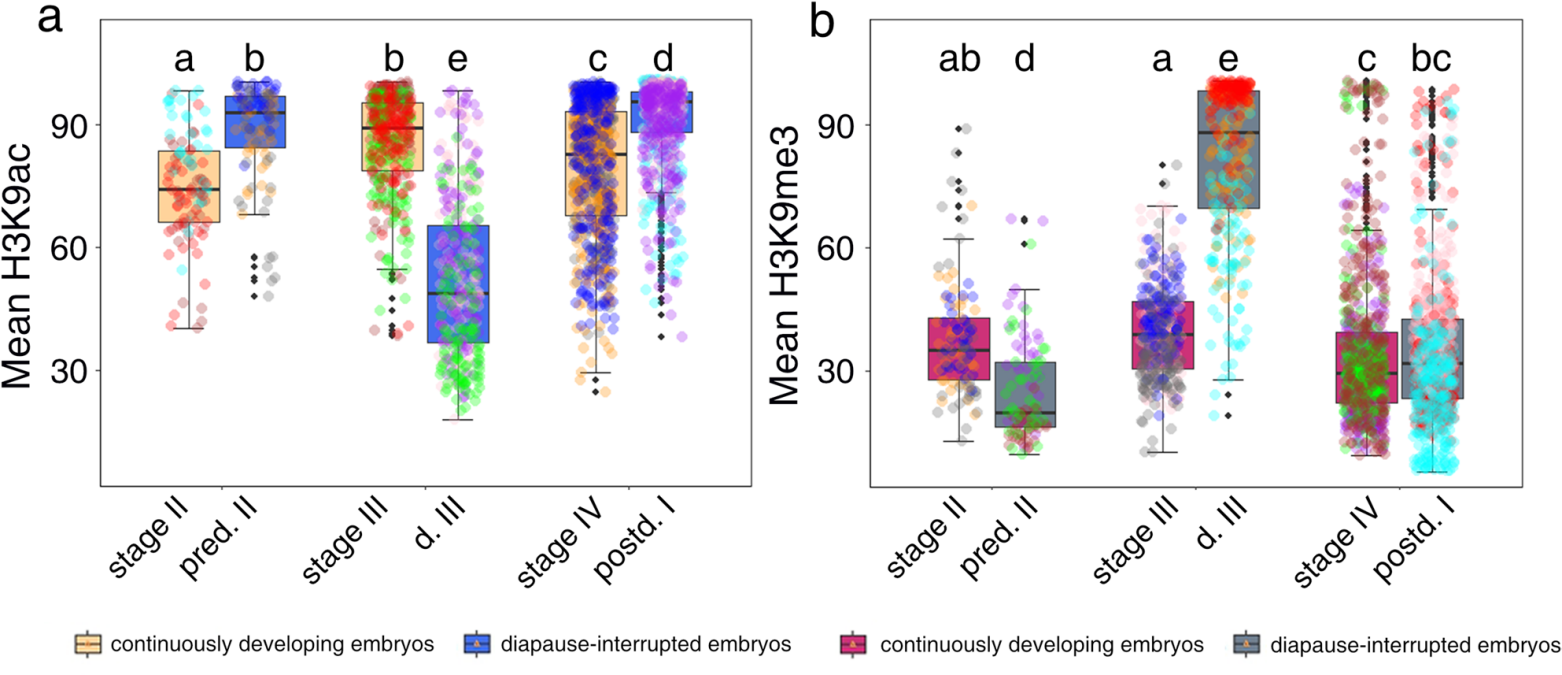
Fluorescence intensity quantification of H3K9ac (**a**) and H3K9me3 (**b**) in *D. magna* continuously developing and diapause-interrupted embryos. The Mean is the average intensity of the pixels in the measured nucleus. Internal lines stand for medians, the top and bottom of each box stand for the third and first quartiles, respectively, upper and lower fence represented the upper and lower whisker (+ 1.5*IQR and − 1.5*IQR). Raw data points were added over boxplots to illustrate the data distribution, with the same color representing data from the same slide. To compare the intensity among groups, Kruskal-Wallis followed by followed by Bonferroni’s post-hoc test. Statistical significance in the hatching rate across groups is indicated by different letters under each bar (*P* ≤ 0.05). Error bars represent standard error (*n* = 3)

The immunolabeling results indicate no distinct differences in the sub-nuclear distribution of H3K9ac and H3K9me3 between diapause-interrupted and continuously developing embryos or across developmental stages (Figs. 2 and 3). However, semi-quantitative analysis reveals significant variations in staining intensity (Fig. 4).

The overall intensity level of H3K9ac exhibits significant variation across both developmental stages and patterns (Kruskal-Wallis test among all experimental groups: H (5, 2400) = 879.30, *P* < 0.001). During continuous embryogenesis, H3K9ac levels are low at stage II, rise significantly by stage III (*P* < 0.001), then decrease significantly at stage IV (*P* < 0.001), although they remain higher than stage II (*P* = 0.0056) (Fig. 4a). In diapause-interrupted embryos, H3K9ac levels in pre-diapause II are significantly higher than those in stage II continuously developing embryos. Upon entering diapause, H3K9ac levels decrease significantly at diapause III, being lower than in pre-diapause II (*P* < 0.001) and stage II and stage III continuously developing embryos (*P* < 0.001). After diapause, during resumed development, H3K9ac levels significantly increase at post-diapause I, exceeding levels in pre-diapause II (*P* = 0.0288), diapause III (*P* < 0.001), and stage IV continuously developing embryos (*P* < 0.001) (Fig. 4a).

Similarly, the overall intensity level of H3K9me3 displays dynamic changes dependent on developmental context (Kruskal-Wallis test among all experimental groups: H (5, 2400) = 771.58, *P* < 0.001). H3K9me3 levels remain steady between stages II and III in continuously developing embryos but drop significantly at stage IV compared to stage II (*P* = 0.0018) and stage III (*P* < 0.001) (Fig. 4b). In diapause-interrupted embryos, H3K9me3 levels at pre-diapause II are significantly lower than in stage II continuously developing embryos (*P* < 0.001). Upon entering diapause, H3K9me3 levels increase significantly at diapause III compared to pre-diapause II (*P* < 0.001) and stages II/III (*P* < 0.001). When development resumes, H3K9me3 levels significantly decrease at post-diapause I, becoming lower than levels at pre-diapause II and diapause III (*P* < 0.001) but comparable to those at stage IV (Fig. 4b).

### *kat2a* and *suv39h1* expression levels during embryonic development in *D. magna* continuously developing and diapause-interrupted embryos

**Fig. 5:**
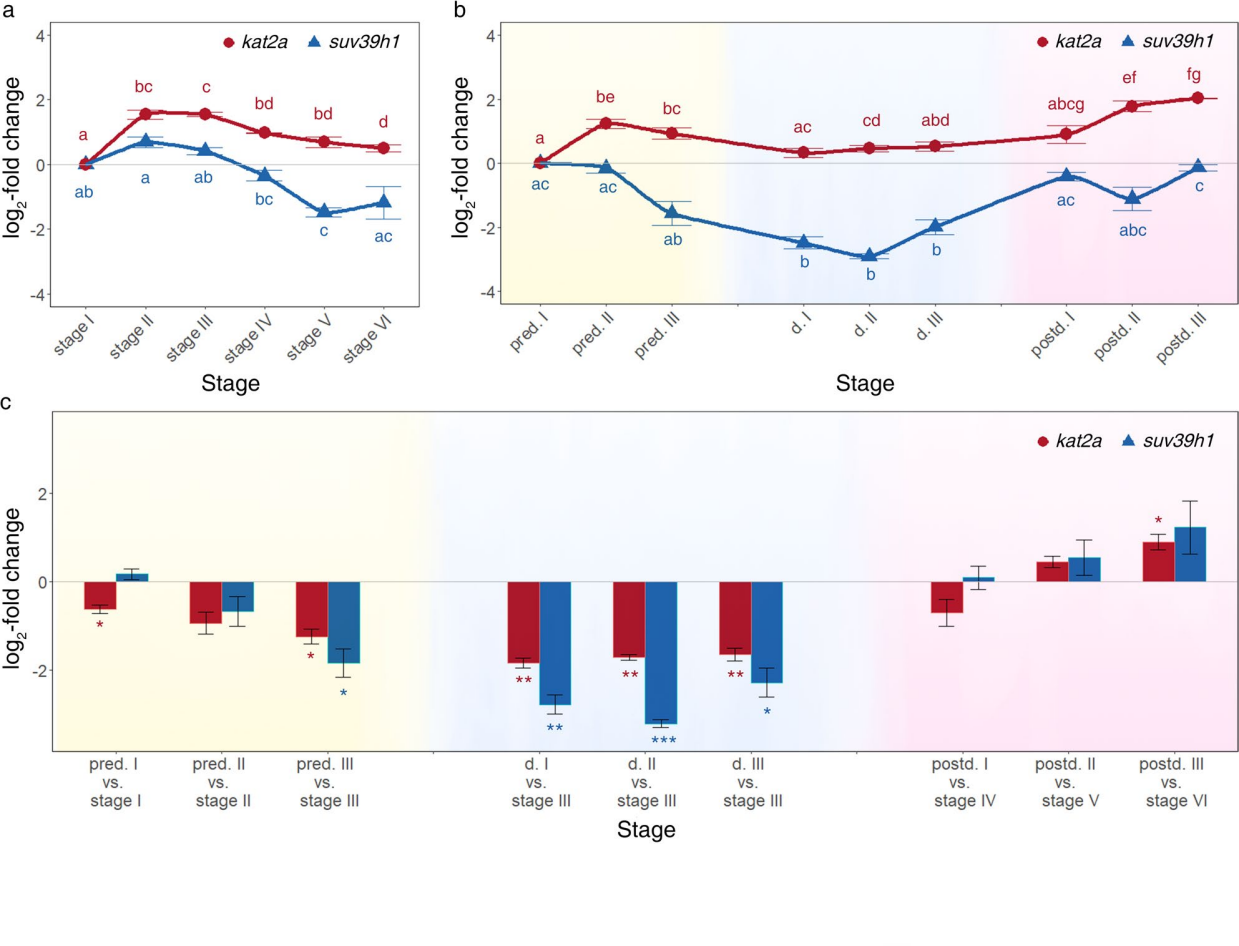
Relative fold change (in log2 scale) in transcript level of *kat2a* and *suv39h1*. *kat2a* and *suv39h1* during embryogenesis of continuously developing embryos (**a**) and diapause-interrupted embryos (**b**). Displayed is expression at each developmental stage relative to the first stage (timing of ovulation). Bars with common letters are not significantly different (*P* ≤ 0.05). Comparison of gene expression of *kat2a* and *suv39h1* (in log2 scale) between continuously developing and diapause-interrupted embryos (**c**). Error bars represent standard error (*n* = 3). P-values are indicated by asterisks: *P ≤ 0.05, **P ≤ 0.01, ***P ≤ 0.001

In continuously developing embryos, the *kat2a* transcript level increases directly after ovulation (stage I) and decreases upon stage IV (Fig. 5a). Similar to the expression in continuously developing embryos, the *kat2a* transcript level increases after ovulation (pre-diapause I) in diapause-interrupted embryos, the transcript level remains high during the pre-diapause phase (pre-diapause II and pre-diapause III). When entering diapause *kat2a* transcript level decreases slightly (diapause I). Its level remains low when embryonic development resumes (post-diapause I) and increases during the subsequent post-diapause development (post-diapause II and post-diapause III) (Fig. 5b). In comparison to the equivalent stages in continuously developing embryos, pre-diapause and diapause embryos have a lower *kat2a* transcript level. The level in post-diapause embryos equals or is higher than the equivalent stages in continuously developing embryos (Fig. 5c).

In continuously developing embryos, *suv39h1* transcript levels show no significant variation across most examined developmental timepoints, with the only exception being a significant decrease at stage V compared to stage I (*P* = 0,0086, Fig. 5a). In diapause-interrupted embryos, the *suv39h1* transcript level remains constant upon ovulation and decreases when entering diapause. Once embryonic development resumes, the *suv39h1* transcript level increases significantly (post-diapause I) and remains constant during the subsequent post-diapause development (Fig. 5b).

In comparison to the equivalent stages in continuously developing embryos, pre-diapause embryos (pre-diapause I and pre-diapause II) exhibit similar levels of *suv39h1* transcripts. Upon late pre-diapause phase (pre-diapause III), the *suv39h1* transcript level is lower in diapause-interrupted embryos than in continuously developing embryos. Upon termination of diapause, *suv39h1* transcript levels during post-diapause development match those in continuously developing embryos at equivalent stages (Fig. 5c).

## Discussion

### Acetylation and methylation at H3K9 in continuously developing embryos

The early stages of normal embryonic development typically rely on maternal resources, with the activation of the embryo’s own gene transcription occurring only after a certain time point [38, 79]. Depending on the species, H3K9ac levels increase either before or after the onset of early transcription (mid-blastula transition) [6, 30, 75], to loosen chromatin and promote transcription. In *Daphnia* continuously developing embryos, H3K9ac signals are clearly observed at stage II (10 h post-ovulation). Although the precise onset of active embryonic transcription in *Daphnia* remains undefined, the presence of H3K9ac signals suggests that the associated genes are in a transcriptionally accessible state. This pattern coincides with increased expression of the acetyltransferase gene *kat2a* during the transition to stage II.

Our previous research has shown that at stage III (15 h after ovulation), although the cell division rate remains high, the acceleration of division begins to slow down [7]. This reflects changes in the cell cycle duration, particularly the extension of interphase. Since interphase is generally associated with increased transcriptional activity, we hypothesize that transcription may have begun in the cells at stage III. The overall increase in H3K9ac signal intensity observed in this study may support the enhancement of transcriptional activity. Moreover, the sustained high transcript levels of *kat2a* from stage II to stage III may underlie the observed increase in H3K9ac, driving the transcriptional activation of H3K9-related genes.

At stage IV, the appearance of abdominal appendages marks the onset of cellular differentiation. We observed a decline in H3K9ac signal intensity, consistent with the differentiation-related reduction of H3K9ac reported in other studies [27, 37, 82]. This decrease indicates a restriction in the expression of H3K9ac-associated genes. However, this decline may be accompanied by a reprogramming of H3K9ac pattern, wherein genes that previously lacked acetylation at H3K9 might now acquire it, and vice versa. Such reprogramming could cause increased expression of cell type-specific genes, despite the overall decrease in H3K9ac levels.

### Methylation at H3K9 in continuously developing embryos

In contrast to H3K9 acetylation, which promotes transcriptional activity, the trimethylation of the H3K9 site is linked to transcriptional repression. H3K9me3 signals are also detected at stage II, suggesting potential chromatin reprogramming that establishes new gene expression programs for early embryonic development [15, 84, 88, 92], likely repressing unnecessary genes to activate essential ones for further development. While H3K9me3 signals are detected at stage II, the transcript level of *suv39h1* gene, which encodes the H3K9 methyltransferase, showed only minor changes from stage I to stage II. It is possible that the *suv39h1* gene is rapidly transcribed for a short period and then quickly returns to baseline levels (discussed later). Alternatively, since stage II is still an early stage of embryonic development, the embryo may rely on maternal SUV39h1 resources to establish this mark.

At stage III, the overall H3K9me3 intensities and transcript levels of *suv39h1* remain constant. We suggest that H3K9me3 and *suv39h1*, by maintaining the repressive state of certain genes, are involved in preparing the embryo to initiate specific gene expression patterns.

The decrease in overall H3K9ac signal at stage IV is not accompanied by an increase in overall H3K9me3 intensities. In fact, our results show that even though some cells exhibit high H3K9me3 signals, the intensity of the majority remains relatively low. H3K9me3-dependent heterochromatin is anticipated to be progressively established and enhanced throughout embryonic development [85]. Even though a significant increase in overall H3K9me3 levels has not yet been observed at stage IV, this does not eliminate the possibility of reprogramming of H3K9me3 patterns. Such reprogramming may be crucial for regulating gene expressions necessary for development, thereby supporting cell differentiation and other critical developmental processes. Whether overall H3K9me3 levels will significantly increase in subsequent stages remains to be investigated. Currently, *suv39h1* genes have been studied mainly in mammalian and zebrafish embryonic development, where they play a crucial role [4, 60, 64], though their expression appears to be transient. In mouse embryos, *suv39h1* transcript levels peak around E10.5, decline through postnatal development, and become nearly absent [55]. In ovine embryos, *suv39h1* gene expression rises from the 2–7 cell stage, peaks during the morula stage, and declines to 2–7 cell stage levels at the blastocyst stage [78]. These suggest a specific temporal role of *suv39h1* during embryogenesis. During the continuous development of *Daphnia*, a significant increase in *suv39h1* expression was not observed at any examined developmental stage. Whether *suv39h1* undergoes one or multiple brief periods of high expression to support histone methylation and whether *suv39h1* is indispensable for *Daphnia* embryogenesis remains to be determined by future research.

Based on the results of H3K9ac and H3K9me3, along with the two genes encoding the enzymes that regulate these modifications, we hypothesize that H3K9 modifications are involved in normal embryogenesis by influencing processes such as chromatin remodelling, transcription regulation, and cellular differentiation. Building on this, we now discuss their potential role in regulating diapause process in diapause-destined embryos of *Daphnia*.

### Acetylation at H3K9 in diapause-destined embryos

At pre-diapause II, the overall H3K9ac level is significantly elevated compared to the corresponding stage in continuously developing embryos (stage II). This elevation, along with our previous findings of an extended cell cycle duration during the pre-diapause phase [7], suggests a higher transcriptional possibility during *Daphnia* pre-diapause phase. High expression of specific genes observed during the pre-diapause phase in both *Daphnia* and other species supports our hypothesis that elevated H3K9ac levels are associated with the activation of specific genes during this phase [8, 16, 47, 67, 76]. Even though higher H3K9ac intensities are observed during pre-diapause, this is not reflected in the *kat2a* transcript level. While there is a rise in *kat2a* upon ovulation, it equals the level observed in stage II of continuously developing embryos. This may indicate a strategy of optimizing resources for the diapause developmental program. In contrast to continuous embryonic development, the primary goal of the pre-diapause phase is to prepare the demands during diapause. Therefore, to optimize resource and energy usage, *kat2a*-encoded acetyltransferases may more specifically target histone sites associated with the diapause developmental program. This may facilitate the expression of key genes required for the pre-diapause phase, resulting in higher overall H3K9ac intensity level in pre-diapausing embryos compared to continuously developing embryos, despite similar *kat2a* transcript level.

During diapause phase, despite the overall arrest of growth and development and the widespread suppression of gene expression, cells maintain sustained gene expression within a defined rage [17, 62, 68, 73, 74]. Our results explain this from the perspective of histone modification. We observe a significant reduction in overall H3K9ac intensity level at diapause III compared to both continuously developing and pre-diapause phases, indicating depressed expressions of H3K9 associated genes. While most cells exhibit very low H3K9ac levels during diapause phase, the high H3K9ac levels observed in a few cells suggest that these cells maintain localized regions of accessible chromatin. This accessible state is likely associated with retained transcriptional potential. *kat2a* transcript level significantly reduces compared to the continuously developing stage but only slightly relative to the pre-diapause phase. We hypothesize that *kat2a* needs to be maintained at a basal level to support the high H3K9ac levels in a few cells, which is crucial for activating the expression of essential genes necessary for the diapause phase. Moreover, maintaining a certain level of *kat2a*, known to affect cell survival [19, 39], is likely vital for the viability of *Daphnia* diapausing embryos throughout the long diapause phase.

At the beginning of resurrection (post-diapause I), the elevated H3K9ac signal suggests the reactivation of chromatin regions with relaxed chromatin and transcriptional competence, presenting a more accessible epigenetic state that enable recovery of gene expression. In fact, H3K9ac level is even higher than at the equivalent stage in continuously developing embryos. This indicates a more extensive opening of chromatin at H3K9ac marked regions, which we hypothesize is required to establish a heightened transcriptionally permissive environment upon diapause termination. However, the increased H3K9ac intensity level in post-diapause I embryos does not come with a significant increase in the *kat2a* transcripts level. Whether other histone acetyltransferases will mediate the H3K9ac enrichment, or whether sufficient *kat2a* left over from the diapause phase can be utilized once diapause ends require additional experimental evidence.

### Methylation at H3K9 in diapause-destined embryos

In contrast to the relatively stable expression pattern of H3K9me3 and *suv39h1* observed in non- diapausing embryos, the two elements exhibit a distinct expression pattern in diapause-destined embryos. The overall intensity level of H3K9me3 is significantly lower at pre-diapause II compared to stage II continuously developing embryos. Combined with the higher H3K9ac level, this supports the hypothesis that genes associated with H3K9 sites are more easily transcribed during the pre-diapause phase, resulting in increased expression of genes specific to diapause developmental program. Similar to continuously developing embryos, we did not detect an increase in *suv39h1* at pre-diapause II. Given the role of *suv39h1* in epigenetic reprogramming [4, 60, 64], we hypothesize that *suv39h1* remains crucial during the pre-diapause phase. However, whether the diapause-interrupted embryo obtains sufficient SUV39h1 enzymes from maternal source or if there is a brief peak in *suv39h1* expression between pre-diapause I and pre-diapause II remain unclear.

The overall intensity of H3K9me3 increases significantly at diapause III. Coupled with the low level of H3K9ac, this points to a localized compaction of chromatin at regions marked by these modifications. This compacted state likely both contributes to and is reinforced by the reduced transcriptional activity of associated genes. Although the overall H3K9me3 levels are relatively high during diapause, we also observed cells with lower H3K9me3 intensity. We hypothesize that cells exhibiting low H3K9me3 levels are simultaneously associated with high H3K9ac levels, and vice versa. If this holds true, it may indicate that during diapause, a minority of cells maintain the transcriptional potential of genes marked by high H3K9ac and low H3K9me, while the majority remain in a dormant state. Despite the significant increase in H3K9me3 levels, *suv39h1* transcript levels decrease significantly. We hypothesize that this pattern results from a two-phase process. An initial establishment phase, potentially driven by a transient peak of *suv39h1* expression or maternal SUV39H1 protein, is followed by a long-term maintenance phase. Once established, the intrinsic kinetic stability of H3K9me3, evidenced by its slow turnover and long half-life [50, 89], reduces the dependency on sustained methyltransferase activity. This model resolves the paradoxical observation of rising H3K9me3 levels alongside declining *suv39h1* expression by establishing a direct link between the mark’s inherent stability and its reduced maintenance demands. Such a mechanism is highly consistent with the energy conservation strategy of diapause. Furthermore, while H3K9me3 stability lessens the core maintenance demand, the evolutionary conservation of histone modifiers suggests that other enzymes, such as G9a or SETDB1, could participate in fine-tuning the repression landscape [46, 57]. Their expression is likewise expected to be low or intermittent.

At postdiapause I, the rise in H3K9ac intensity level is coupled with a significant reduction in H3K9me3 intensity levels, reflecting a remodeling of H3K9 marked chromatin into a more permissive state. This enhances accessibility to facilitate the gene transcription necessary for the resumption of development. Concurrently, *suv39h1* transcript levels increase and return to the pre-diapause level. While the increase in *suv39h1* may seem contradictory to the decrease in H3K9me3, it likely underscores the role of *suv39h1* in terminating diapause and facilitating post-diapause development by reprogramming H3K9me3 patterns.

### Histone modifications and diapause regulation

Based on previous findings we hypothesized a pivotal role of histone modifications in diapause regulation (Table 1), and examined the H3K9 patterns in diapause-destined embryos of *Daphnia*. Our findings reveal a heterogeneous distribution of H3K9 modifications, marked by the co-existence of a repressive state (high H3K9me3/low H3K9ac) in most cells with an active state (high H3K9ac/low H3K9me3) in a minority. This finding aligns with the established concept that histone modifications can mediate highly specific regulatory outcomes [2, 40, 69]. The presence of such a distinct cell population in *Daphnia* diapausing embryos indicates that certain cell types or subpopulations maintain transcriptional competence for H3K9-related genes. Furthermore, transcriptional regulation during diapause may occur with high specificity at the genomic level. In many diapausing species, transcriptional activity persists in a limited gene set sustaining vital function, contrasting with genome-wide silencing [16, 44]. Hence, we propose that in *Daphnia*, the high H3K9ac and low H3K9me3 levels in specific cells create a permissive chromatin environment that is conducive to the expression of genes critical for diapause. These may include genes related to stress response, circadian clock, and epigenetic control, which are likely to continue being transcribed in certain cells during diapaus period [17, 62, 68, 73, 74]. We propose that the maintenance of transcriptional potential at H3K9-marked loci in a minority of cells serves as a strategic energy-saving mechanism for diapausing embryos, sustaining essential functions while priming a rapid response to hatching signals.

In addition to the specificity of H3K9 modifications, the cross-talk between different histone modifications is critical for understanding gene regulation during diapause development [22, 53, 91]. The observed reconfiguration of the H3K9 landscape in diapausing *Daphnia* embryos may not only contribute to transcriptional suppression at target loci but may also engage in crosstalk with other histone modifications. For instance, elevated H3K9me3 is positioned to suppress the establishment of transcription-promoting marks such as H3K4 methylation. Conversely, the reduction in H3K9ac likely impairs its synergistic relationship with activators such as H3K4me3, thereby compromising the coordinated activation of related genes [48, 83]. Furthermore, the increase in H3K9me3 and decrease in H3K9ac may be functionally linked to the significant reduction or absence of the mitotic marker H3S10 phosphorylation during the growth-arrested diapause period [7, 10]. Given that H3S10ph is a known negative regulator of H3K9 methylation and exhibits positive crosstalk with H3K9ac [65], its absence provides a plausible mechanism for sustaining the observed high H3K9me3 and low H3K9ac levels in diapausing *Daphnia* embryos. The H3K9-centered changes observed throughout *Daphnia* diapause development may be part of a broader regulator network, potentially coordinating subsequent developmental and physiological transitions by fine-tuning key gene expression. Our work on H3K9 modifications lays the groundwork for studying *Daphnia* diapause regulation. Unraveling the functional interplay between virous histone marks and their integration with other epigenetic regulators will be a crucial objective for future studies.

## Conclusion

Our study compares changes in H3K9 modifications in diapause-interrupted and continuously developing embryos of *Daphnia*. We observed a significant decrease in H3K9ac and an increase in H3K9me3 during diapause, suggesting a more repressive environment at chromatin regions marked by H3K9. This repressive chromatin landscape likely fosters a transcriptionally repressive environment that contributes to the cessation of growth and development.

*kat2a* is highly expressed during active embryonic development but declines during diapause. However, its sustained basal expression is likely crucial for maintaining H3K9ac in certain cells, thereby sustaining the expression of essential genes required for survival during this dormant period. Even though the link between *suv39h1* and elevated H3K9me3 levels during diapause remains uncertain, its increased expression after diapause suggests it may participate in chromatin reprogramming as embryonic development resumes. This finding indicates the role of H3K9 modifications in regulating chromatin structure, thereby influencing the diapause process in diapause-destined embryos of *Daphnia*.

## Supplementary Information

Supplementary material 1

Supplementary material 2.

## Data Availability

All data is included in this article.
